# Charge-related topological index – various chemoinformatics applications

**DOI:** 10.1186/1758-2946-6-S1-P7

**Published:** 2014-03-11

**Authors:** Nikolay T Kochev, Ivan Bangov, Emil Petrov, Marina Moskovkina, Borislav Stoyanov

**Affiliations:** 1 University of Plovdiv, Department of Analytical Chemistry and Computer Chemistry, University of Plovdiv, 24 Tsar Assen St., Plovdiv, Bulgaria; 2 Faculty of Natural Sciences, Konstantin Preslavski Shumen University, 115 Universitetska Str., Shumen, Bulgaria; 3 Department of Computer Informatics Faculty of Mathematics and Informatica, Konstantin Preslavski University of Shumen, 115 Universitetska Str., Shumen, Bulgaria

## 

We present several useful applications of the CTI index in the context of various chemoinformatics tasks. **C**harge-related **T**opological **I**ndex (CTI) was introduced initially by Bangov for solving the problem of 2D structure isomorphism within the computer-assisted structure generation from a gross formula [1]. CTI is a real number defined as a sum over all atom pairs:  where *D_ij_* is the inter-atomic topological distance and  is a local atom index (a characteristics of atom i) calculated from the atom valence, *L_0,i_*, the number of hydrogen atoms attached to atom, *N_H,i_*, and *q_i_* which is the corresponding charge density. The partial charges are computed by the topological empirical method of Gasteiger-Marsili [2] calculated with a fixed number of iterations. The CTI index could be used for 3D structure and conformer perception [3] in the following form:  where *R_ij_* is the geometrical distance (in the latter case the charges could be also obtained from quantum chemistry programs on a semi-empirical level). Respectively, *L_i_* values could be employed to the perception of the structure symmetry and topological equivalence. In our latest study we proved the capabilities of CTI index for perception of duplicated structures in large structure collections [4]. It is shown that the CTI index can be safely used for a quick perception of the duplicated structures in large databases in a very fast identity (full structure) search. CTI index with a precision of 10 digits after decimal point can be used in databases with millions of compounds. It has been also shown that CTI can be used as a useful descriptor well describing both the structure branching and some electronic properties [3].
